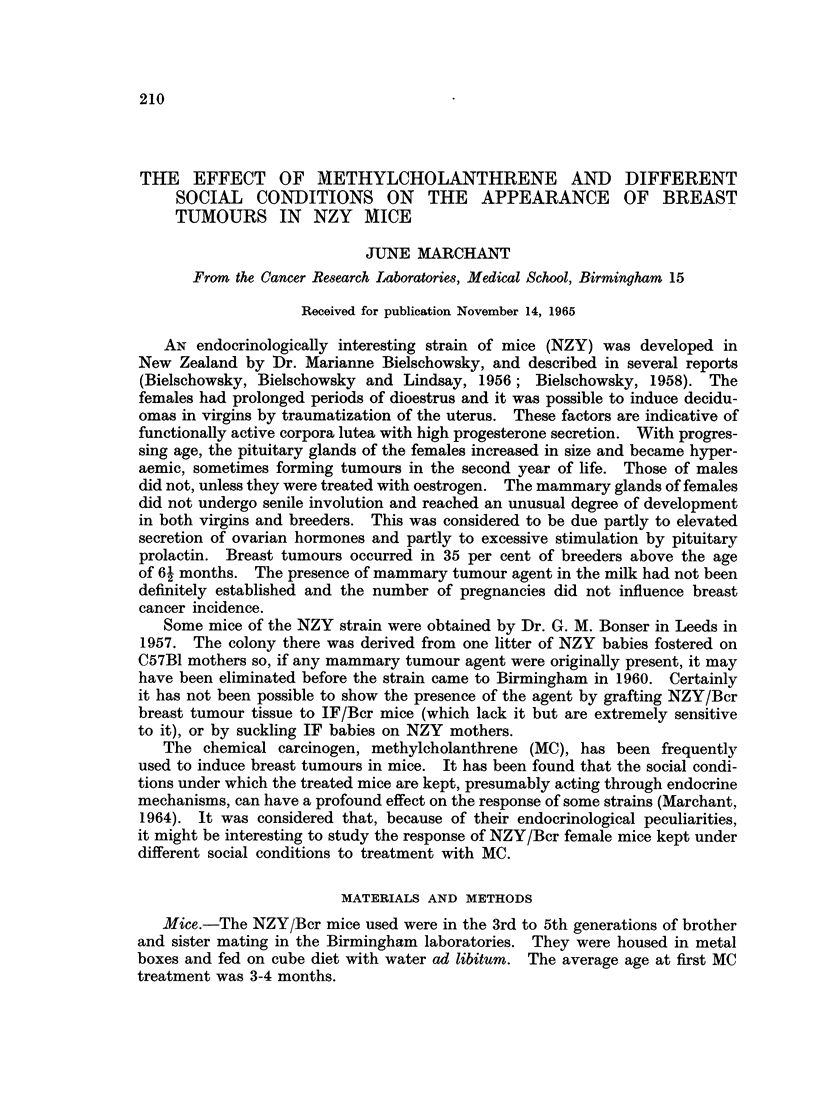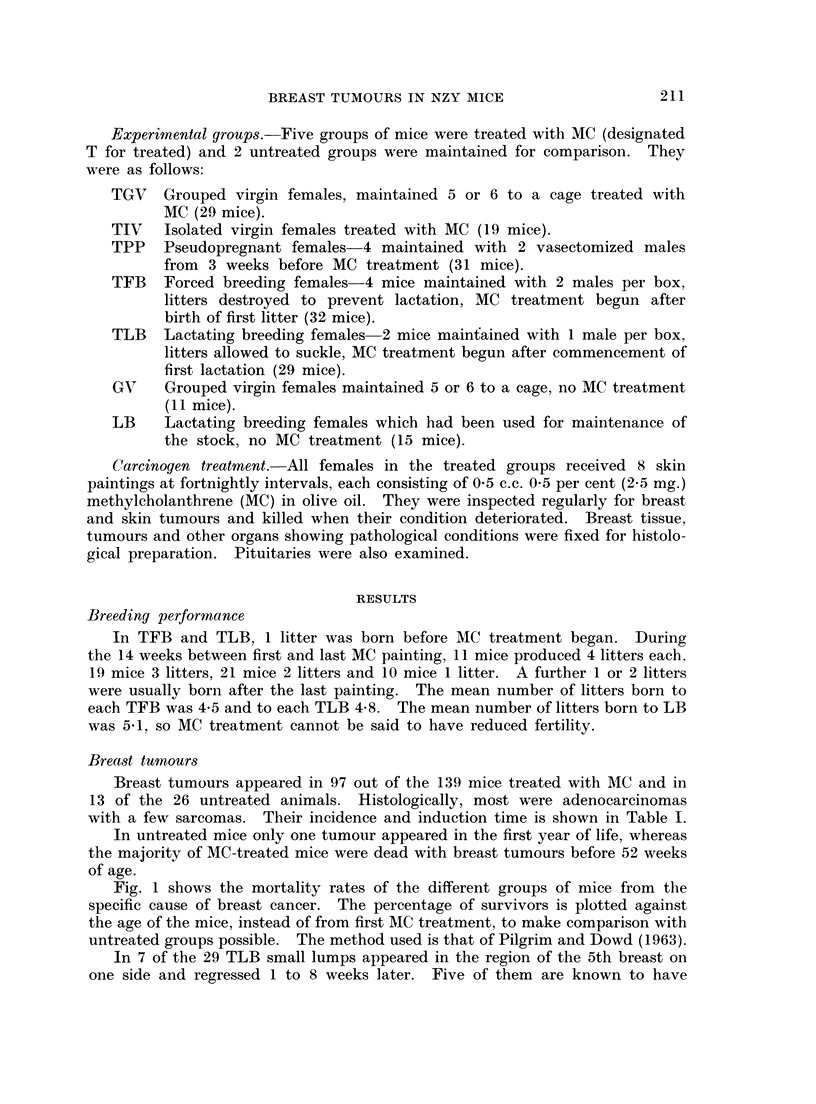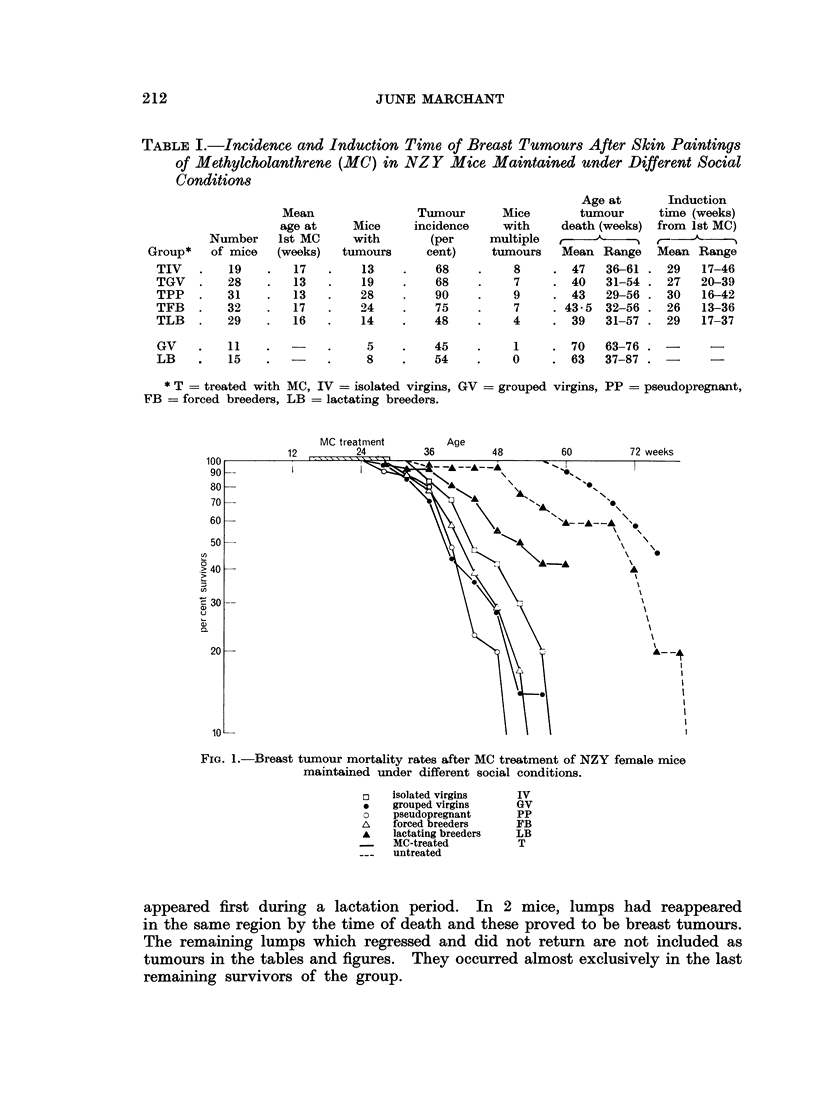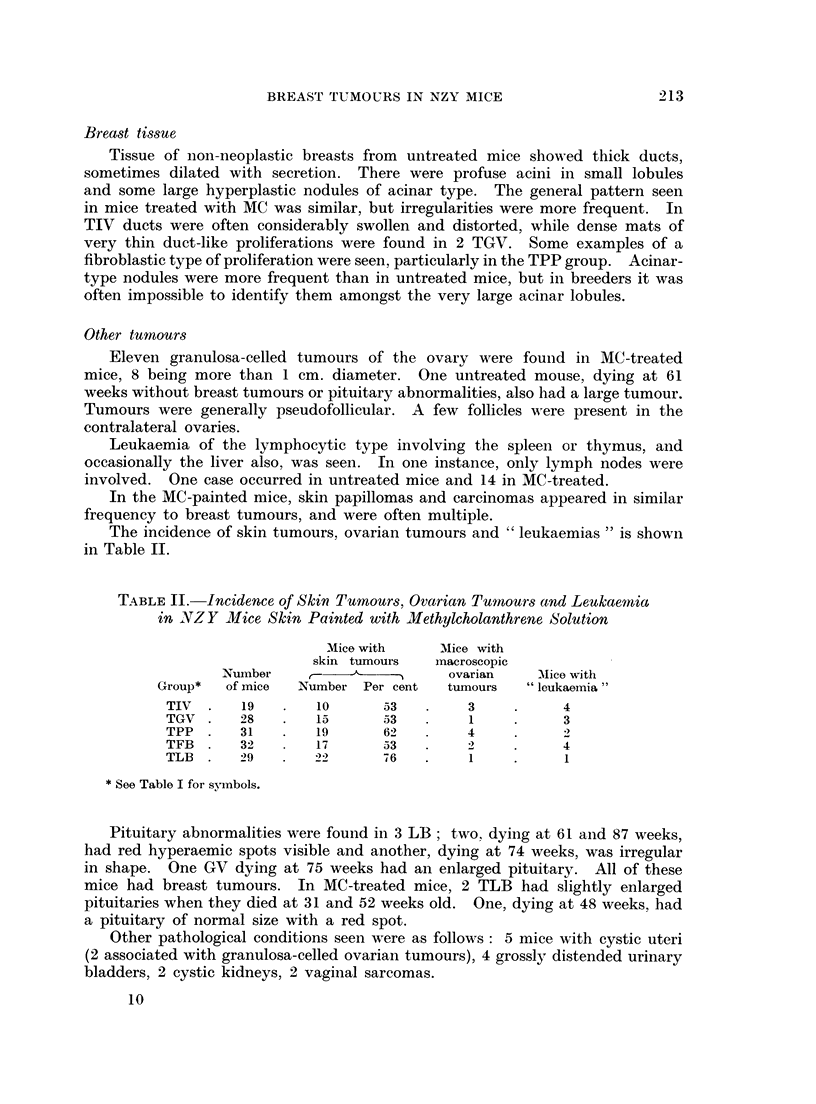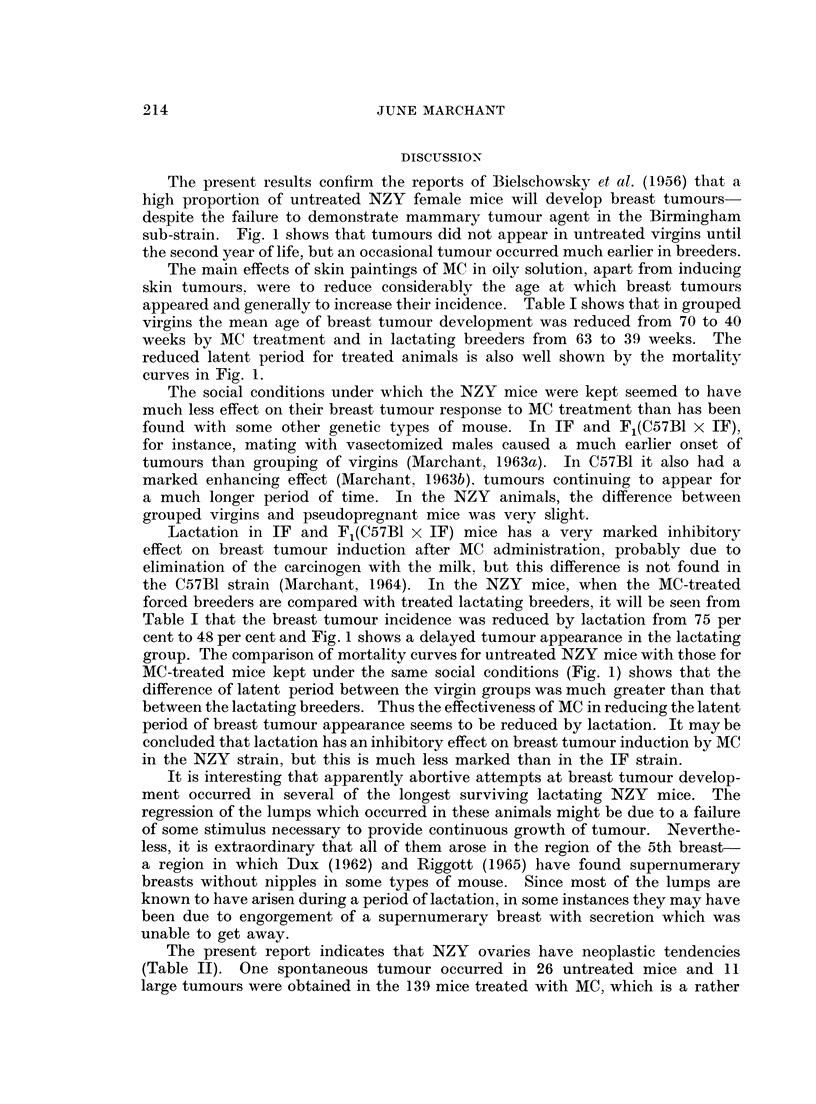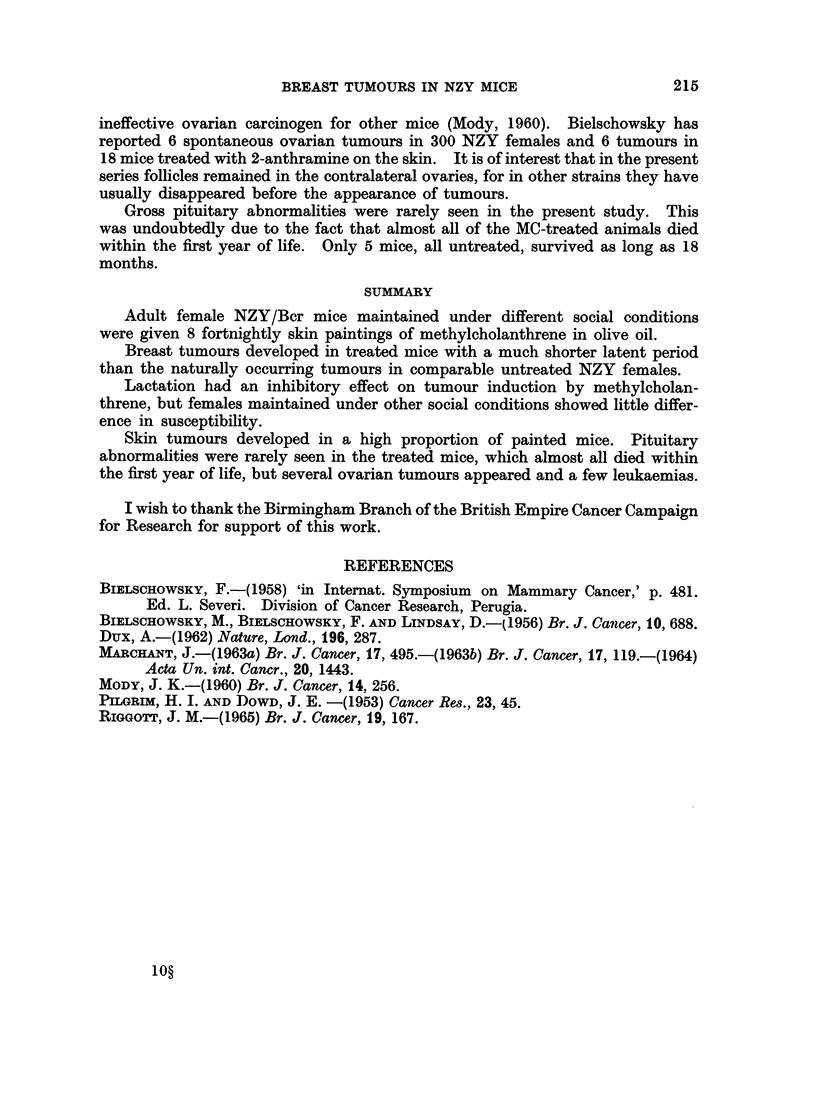# The effect of methylcholanthrene and different social conditions on the appearance of breast tumours in NZY mice.

**DOI:** 10.1038/bjc.1966.25

**Published:** 1966-03

**Authors:** J. Marchant


### THE EFFECT OF METHYLCHOLANTHRENE AND DIFFERENT SOCIAL CONDITIONS ON THE APPEARANCE OF BREAST TUMOURS IN NZY MICE

JUNE MARCHANT

*From the Cancer Research Laboratories, Medical School, Birmingham* 15

Received for publication November 14, 1965

An endocrinologically interesting strain of mice (NZY) was developed in New Zealand by Dr. Marianne Bielschowsky, and described in several reports (Bielschowsky, Bielschowsky and Lindsay, 1956; Bielschowsky, 1958). The females had prolonged periods of dioestrus and it was possible to induce deciduomas in virgins by traumatization of the uterus. These factors are indicative of functionally active corpora lutea with high progesterone secretion. With progressing age, the pituitary glands of the females increased in size and became hyperaemic, sometimes forming tumours in the second year of life. Those of males did not, unless they were treated with oestrogen. The mammary glands of females did not undergo senile involution and reached an unusual degree of development in both virgins and breeders. This was considered to be due partly to elevated secretion of ovarian hormones and partly to excessive stimulation by pituitary prolactin. Breast tumours occurred in 35 per cent of breeders above the age of 6½ months. The presence of mammary tumour agent in the milk had not been definitely established and the number of pregnancies did not influence breast cancer incidence.

Some mice of the NZY strain were obtained by Dr. G. M. Bonser in Leeds in 1957. The colony there was derived from one litter of NZY babies fostered on C57Bl mothers so, if any mammary tumour agent were originally present, it may have been eliminated before the strain came to Birmingham in 1960. Certainly it has not been possible to show the presence of the agent by grafting NZY/Bcr breast tumour tissue to IF/Bcr mice (which lack it but are extremely sensitive to it), or by suckling IF babies on NZY mothers.

The chemical carcinogen, methylcholanthrene (MC), has been frequently used to induce breast tumours in mice. It has been found that the social conditions under which the treated mice are kept, presumably acting through endocrine mechanisms, can have a profound effect on the response of some strains (Marchant, 1964). It was considered that, because of their endocrinological peculiarities, it might be interesting to study the response of NZY/Bcr female mice kept under different social conditions to treatment with MC.

### MATERIALS AND METHODS

*Mice.*—The NZY/Bcr mice used were in the 3rd to 5th generations of brother and sister mating in the Birmingham laboratories. They were housed in metal boxes and fed on cube diet with water *ad libitum.* The average age at first MC treatment was 3-4 months.

*Experimental groups.*—Five groups of mice were treated with MC (designated T for treated) and 2 untreated groups were maintained for comparison. They were as follows:

TGV Grouped virgin females, maintained 5 or 6 to a cage treated with MC (29 mice).
TIV Isolated virgin females treated with MC (19 mice).
TPP Pseudopregnant females—4 maintained with 2 vasectomized males from 3 weeks before MC treatment (31 mice).
TFB Forced breeding females—4 mice maintained with 2 males per box, litters destroyed to prevent lactation, MC treatment begun after birth of first litter (32 mice).
TLB Lactating breeding females—2 mice maintained with 1 male per box, litters allowed to suckle, MC treatment begun after commencement of first lactation (29 mice).
GV Grouped virgin females maintained 5 or 6 to a cage, no MC treatment (11 mice).
LB Lactating breeding females which had been used for maintenance of the stock, no MC treatment (15 mice).

*Carcinogen treatment.*—All females in the treated groups received 8 skin paintings at fortnightly intervals, each consisting of 0·5 c.c. 0·5 per cent (2·5 mg.) methylcholanthrene (MC) in olive oil. They were inspected regularly for breast and skin tumours and killed when their condition deteriorated. Breast tissue, tumours and other organs showing pathological conditions were fixed for histological preparation. Pituitaries were also examined.

### RESULTS

### *Breeding performance*

In TFB and TLB, 1 litter was born before MC treatment began. During the 14 weeks between first and last MC painting, 11 mice produced 4 litters each, 19 mice 3 litters, 21 mice 2 litters and 10 mice 1 litter. A further 1 or 2 litters were usually born after the last painting. The mean number of litters born to each TFB was 4·5 and to each TLB 4·8. The mean number of litters born to LB was 5·1, so MC treatment cannot be said to have reduced fertility.

### *Breast tumours*

Breast tumours appeared in 97 out of the 139 mice treated with MC and in 13 of the 26 untreated animals. Histologically, most were adenocarcinomas with a few sarcomas. Their incidence and induction time is shown in Table I.

In untreated mice only one tumour appeared in the first year of life, whereas the majority of MC-treated mice were dead with breast tumours before 52 weeks of age.

Fig. 1 shows the mortality rates of the different groups of mice from the specific cause of breast cancer. The percentage of survivors is plotted against the age of the mice, instead of from first MC treatment, to make comparison with untreated groups possible. The method used is that of Pilgrim and Dowd (1963).

In 7 of the 29 TLB small lumps appeared in the region of the 5th breast on one side and regressed 1 to 8 weeks later. Five of them are known to have

TABLE I.—*Incidence and Induction Time of Breast Tumours After Skin Paintings of Methylcholanthrene (MC) in NZY Mice Maintained under Different Social Conditions*

| Group* | Number of mice | Mean age at 1st MC (weeks) | Mice with tumours | Tumour incidence (per cent) | Mice with multiple tumours | Age at tumour death (weeks) | | Induction time (weeks) from 1st MC | |
|---|---|---|---|---|---|---|---|---|---|
| | | | | | | Mean | Range | Mean | Range |
| TIV | 19 | 17 | 13 | 68 | 8 | 47 | 36–61 | 29 | 17–46 |
| TGV | 28 | 13 | 19 | 68 | 7 | 40 | 31–54 | 27 | 20–39 |
| TPP | 31 | 13 | 28 | 90 | 9 | 43 | 29–56 | 30 | 16–42 |
| TFB | 32 | 17 | 24 | 75 | 7 | 43·5 | 32–56 | 26 | 13–36 |
| TLB | 29 | 16 | 14 | 48 | 4 | 39 | 31–57 | 29 | 17–37 |
| GV | 11 | — | 5 | 45 | 1 | 70 | 63–76 | — | — |
| LB | 15 | — | 8 | 54 | 0 | 63 | 37–87 | — | — |

* T = treated with MC, IV = isolated virgins, GV = grouped virgins, PP = pseudopregnant, FB = forced breeders, LB = lactating breeders.

FIG. 1.—Breast tumour mortality rates after MC treatment of NZY female mice maintained under different social conditions.

| | | |
|---|---|---|
| □ | isolated virgins | IV |
| ● | grouped virgins | GV |
| ○ | pseudopregnant | PP |
| △ | forced breeders | FB |
| ▲ | lactating breeders | LB |
| — | MC-treated | T |
| --- | untreated | |

appeared first during a lactation period. In 2 mice, lumps had reappeared in the same region by the time of death and these proved to be breast tumours. The remaining lumps which regressed and did not return are not included as tumours in the tables and figures. They occurred almost exclusively in the last remaining survivors of the group.

*Breast tissue*

Tissue of non-neoplastic breasts from untreated mice showed thick ducts, sometimes dilated with secretion. There were profuse acini in small lobules and some large hyperplastic nodules of acinar type. The general pattern seen in mice treated with MC was similar, but irregularities were more frequent. In TIV ducts were often considerably swollen and distorted, while dense mats of very thin duct-like proliferations were found in 2 TGV. Some examples of a fibroblastic type of proliferation were seen, particularly in the TPP group. Acinar-type nodules were more frequent than in untreated mice, but in breeders it was often impossible to identify them amongst the very large acinar lobules.

*Other tumours*

Eleven granulosa-celled tumours of the ovary were found in MC-treated mice, 8 being more than 1 cm. diameter. One untreated mouse, dying at 61 weeks without breast tumours or pituitary abnormalities, also had a large tumour. Tumours were generally pseudofollicular. A few follicles were present in the contralateral ovaries.

Leukaemia of the lymphocytic type involving the spleen or thymus, and occasionally the liver also, was seen. In one instance, only lymph nodes were involved. One case occurred in untreated mice and 14 in MC-treated.

In the MC-painted mice, skin papillomas and carcinomas appeared in similar frequency to breast tumours, and were often multiple.

The incidence of skin tumours, ovarian tumours and " leukaemias " is shown in Table II.

TABLE II.—*Incidence of Skin Tumours, Ovarian Tumours and Leukaemia in NZY Mice Skin Painted with Methylcholanthrene Solution*

| Group* | Number of mice | Mice with skin tumours: Number | Mice with skin tumours: Per cent | Mice with macroscopic ovarian tumours | Mice with " leukaemia " |
|---|---|---|---|---|---|
| TIV | 19 | 10 | 53 | 3 | 4 |
| TGV | 28 | 15 | 53 | 1 | 3 |
| TPP | 31 | 19 | 62 | 4 | 2 |
| TFB | 32 | 17 | 53 | 2 | 4 |
| TLB | 29 | 22 | 76 | 1 | 1 |

* See Table I for symbols.

Pituitary abnormalities were found in 3 LB ; two, dying at 61 and 87 weeks, had red hyperaemic spots visible and another, dying at 74 weeks, was irregular in shape. One GV dying at 75 weeks had an enlarged pituitary. All of these mice had breast tumours. In MC-treated mice, 2 TLB had slightly enlarged pituitaries when they died at 31 and 52 weeks old. One, dying at 48 weeks, had a pituitary of normal size with a red spot.

Other pathological conditions seen were as follows : 5 mice with cystic uteri (2 associated with granulosa-celled ovarian tumours), 4 grossly distended urinary bladders, 2 cystic kidneys, 2 vaginal sarcomas.

### DISCUSSION

The present results confirm the reports of Bielschowsky *et al.* (1956) that a high proportion of untreated NZY female mice will develop breast tumours—despite the failure to demonstrate mammary tumour agent in the Birmingham sub-strain. Fig. 1 shows that tumours did not appear in untreated virgins until the second year of life, but an occasional tumour occurred much earlier in breeders.

The main effects of skin paintings of MC in oily solution, apart from inducing skin tumours, were to reduce considerably the age at which breast tumours appeared and generally to increase their incidence. Table I shows that in grouped virgins the mean age of breast tumour development was reduced from 70 to 40 weeks by MC treatment and in lactating breeders from 63 to 39 weeks. The reduced latent period for treated animals is also well shown by the mortality curves in Fig. 1.

The social conditions under which the NZY mice were kept seemed to have much less effect on their breast tumour response to MC treatment than has been found with some other genetic types of mouse. In IF and $F_1$(C57Bl $\times$ IF), for instance, mating with vasectomized males caused a much earlier onset of tumours than grouping of virgins (Marchant, 1963*a*). In C57Bl it also had a marked enhancing effect (Marchant, 1963*b*), tumours continuing to appear for a much longer period of time. In the NZY animals, the difference between grouped virgins and pseudopregnant mice was very slight.

Lactation in IF and $F_1$(C57Bl $\times$ IF) mice has a very marked inhibitory effect on breast tumour induction after MC administration, probably due to elimination of the carcinogen with the milk, but this difference is not found in the C57Bl strain (Marchant, 1964). In the NZY mice, when the MC-treated forced breeders are compared with treated lactating breeders, it will be seen from Table I that the breast tumour incidence was reduced by lactation from 75 per cent to 48 per cent and Fig. 1 shows a delayed tumour appearance in the lactating group. The comparison of mortality curves for untreated NZY mice with those for MC-treated mice kept under the same social conditions (Fig. 1) shows that the difference of latent period between the virgin groups was much greater than that between the lactating breeders. Thus the effectiveness of MC in reducing the latent period of breast tumour appearance seems to be reduced by lactation. It may be concluded that lactation has an inhibitory effect on breast tumour induction by MC in the NZY strain, but this is much less marked than in the IF strain.

It is interesting that apparently abortive attempts at breast tumour development occurred in several of the longest surviving lactating NZY mice. The regression of the lumps which occurred in these animals might be due to a failure of some stimulus necessary to provide continuous growth of tumour. Nevertheless, it is extraordinary that all of them arose in the region of the 5th breast—a region in which Dux (1962) and Riggott (1965) have found supernumerary breasts without nipples in some types of mouse. Since most of the lumps are known to have arisen during a period of lactation, in some instances they may have been due to engorgement of a supernumerary breast with secretion which was unable to get away.

The present report indicates that NZY ovaries have neoplastic tendencies (Table II). One spontaneous tumour occurred in 26 untreated mice and 11 large tumours were obtained in the 139 mice treated with MC, which is a rather ineffective ovarian carcinogen for other mice (Mody, 1960). Bielschowsky has reported 6 spontaneous ovarian tumours in 300 NZY females and 6 tumours in 18 mice treated with 2-anthramine on the skin. It is of interest that in the present series follicles remained in the contralateral ovaries, for in other strains they have usually disappeared before the appearance of tumours.

Gross pituitary abnormalities were rarely seen in the present study. This was undoubtedly due to the fact that almost all of the MC-treated animals died within the first year of life. Only 5 mice, all untreated, survived as long as 18 months.

SUMMARY

Adult female NZY/Bcr mice maintained under different social conditions were given 8 fortnightly skin paintings of methylcholanthrene in olive oil.

Breast tumours developed in treated mice with a much shorter latent period than the naturally occurring tumours in comparable untreated NZY females.

Lactation had an inhibitory effect on tumour induction by methylcholanthrene, but females maintained under other social conditions showed little difference in susceptibility.

Skin tumours developed in a high proportion of painted mice. Pituitary abnormalities were rarely seen in the treated mice, which almost all died within the first year of life, but several ovarian tumours appeared and a few leukaemias.

I wish to thank the Birmingham Branch of the British Empire Cancer Campaign for Research for support of this work.

### REFERENCES

BIELSCHOWSKY, F.—(1958) 'in Internat. Symposium on Mammary Cancer,' p. 481. Ed. L. Severi. Division of Cancer Research, Perugia.

BIELSCHOWSKY, M., BIELSCHOWSKY, F. AND LINDSAY, D.—(1956) *Br. J. Cancer*, **10**, 688.

DUX, A.—(1962) *Nature, Lond.*, **196**, 287.

MARCHANT, J.—(1963*a*) *Br. J. Cancer*, **17**, 495.—(1963*b*) *Br. J. Cancer*, **17**, 119.—(1964) *Acta Un. int. Cancr.*, **20**, 1443.

MODY, J. K.—(1960) *Br. J. Cancer*, **14**, 256.

PILGRIM, H. I. AND DOWD, J. E. —(1953) *Cancer Res.*, **23**, 45.

RIGGOTT, J. M.—(1965) *Br. J. Cancer*, **19**, 167.